# Ethylformiat: MAK-Begründung, Nachtrag

**DOI:** 10.34865/mb10994d10_2ad

**Published:** 2025-06-30

**Authors:** Andrea Hartwig, MAK Commission

**Affiliations:** 1 Institut für Angewandte Biowissenschaften. Abteilung Lebensmittelchemie und Toxikologie. Karlsruher Institut für Technologie (KIT) Adenauerring 20a, Geb. 50.41 76131 Karlsruhe Deutschland; 2 Ständige Senatskommission zur Prüfung gesundheitsschädlicher Arbeitsstoffe. Deutsche Forschungsgemeinschaft, Kennedyallee 40, 53175 Bonn, Deutschland. Weitere Informationen: Ständige Senatskommission zur Prüfung gesundheitsschädlicher Arbeitsstoffe | DFG

**Keywords:** Ethylformiat, Reizwirkung, Auge, Nase, MAK-Wert, maximale Arbeitsplatzkonzentration, Spitzenbegrenzung, Hautresorption, Analogiebetrachtung, ethyl formiate, irritation, eye, nose, MAK value, maximum workplace concentration, peak limitation, skin absorption, read across

## Abstract

The German Senate Commission for the Investigation of Health Hazards of Chemical Compounds in the Work Area (MAK Commission) summarized and re-evaluated the data for ethyl formate [109-94-4] to derive an occupational exposure limit value (maximum concentration at the workplace, MAK value) considering all toxicological end points. Relevant studies were identified from a literature search. The critical effects of ethyl formate are irritation of the eyes and nose in humans and irritation of the olfactory mucosa in rats; neurotoxic effects were observed in animals at higher concentrations. Based on the lowest observed adverse effect concentration (LOAEC) of 330 ml/m^3^ for irritation in a study with volunteers, a NAEC was extrapolated and the previous MAK value of 100 ml/m^3^ has been retained. This concentration provides protection also against neurotoxic effects. The MAK value of ethyl formate is thus higher than that of its metabolite formic acid (5 ml/m^3^), which is consistent with the analogous situation of ethyl acetate (MAK value 200 ml/m^3^) and acetic acid (MAK value 10 ml/m^3^). The assignment to Peak Limitation Category I with an excursion factor of 1 has been retained. Taking into consideration the data for the metabolites formic acid and ethanol, damage to the embryo or foetus is unlikely if the MAK value of ethyl formate is observed. Ethyl formate is not mutagenic in bacteria or yeasts. No other genotoxicity or carcinogenicity studies are available, but the structure of ethyl formate does not give reason to expect these types of effects. Ethyl formate remains designated with “H” for substances which can be taken up via the skin in toxicologically relevant amounts. There is no structural alert for sensitization.

**Table:** 

**MAK-Wert (1997)**	**100 ml/m^3^ (ppm) ≙ 310 mg/m^3^**
**Spitzenbegrenzung (2000)**	**Kategorie I, Überschreitungsfaktor 1**
	
**Hautresorption (1997)**	**H**
**Sensibilisierende Wirkung**	**–**
**Krebserzeugende Wirkung**	**–**
**Fruchtschädigende Wirkung (1997)**	**Gruppe C**
**Keimzellmutagene Wirkung**	**–**
	
**BAT-Wert**	**–**
CAS-Nr.	109-94-4
Molmasse	74,08 g/mol
Dampfdruck bei 20 °C	266,64 hPa (ECHA 2022)
Siedepunkt bei 978,9 hPa	56,6 °C (ECHA 2022)
Wasserlöslichkeit	71 781,8 mg/l (ECHA 2022)
log K_OW_	1,504 (ECHA 2022)
**1 ml/m^3^ (ppm) ≙ 3,074 mg/m^3^**	**1 mg/m^3^ ≙ 0,325 ml/m^3^ (ppm)**

Seit Erscheinen der Begründung von 1997 (Greim 1997 b) sind zwei Probandenstudien sowie eine 90-Tage-Inhalationsstudie an Ratten publiziert worden, weswegen eine Überprüfung des MAK-Wertes und aller Endpunkte erfolgt sowie die Daten zur Keimzellmutagenität erstmals bewertet werden.

## 1 Allgemeiner Wirkungscharakter

Ethylformiat wird zu Ameisensäure und Ethanol metabolisiert. Bei 100 ml Ethylformiat/m^3^ ist weder mit einer Akkumulation der Metaboliten Ethanol oder Ameisensäure noch mit einer metabolischen Azidose zu rechnen.

Ethylformiat wirkt nicht reizend an der Kaninchenhaut, aber reizend am Kaninchenauge. Beim Menschen werden bei inhalativer Exposition lokale Reizwirkungen an Auge und Nase beschrieben.

In einer 90-Tage-Inhalationsstudie an Ratten kommt es ab 330 ml/m^3^ zu Degenerationen im olfaktorischen Epithel und bei 1320 ml/m^3^ zu Plattenepithelmetaplasien des olfaktorischen Epithels sowie zu verminderter Futteraufnahme, verzögerter Körpergewichtsentwicklung, sowie einer reversiblen Reduktion der lokomotorischen Aktivität.

Zur haut- und atemwegssensibilisierenden Wirkung liegen keine verwertbaren Daten vor.

Ethylformiat ist in Bakterien und Hefen nicht mutagen. Zu den Endpunkten Entwicklungstoxizität und Kanzerogenität liegen zu Ethylformiat keine bzw. keine validen Studien vor.

## 2 Wirkungsmechanismus

Die irritative Wirkung von Ethylformiat wird auf das Hydrolyseprodukt Ameisensäure zurückgeführt (Greim 1997 b).

Die metabolische Azidose beruht auf der Absenkung des pH-Wertes im Blut durch die Entstehung von Ameisen- und Essigsäure (Greim 1997 b).

## 3 Toxikokinetik und Metabolismus

### 3.1 Aufnahme, Verteilung, Ausscheidung

Ethylformiat wird inhalativ und oral resorbiert. Aufgrund der physikalischen Eigenschaften wurde ein dermaler Flux von 0,71 mg/cm^2^ und Stunde berechnet (ECB 2000; Health Council of the Netherlands 2002).

Für eine gesättigte wässrige Lösung berechnen sich mit dem Modell von Fiserova-Bergerova et al. (1990) und dem Algorithmus des IH SkinPerm-Modells (Tibaldi et al. 2014) Fluxe von 7200 bzw. 734 µg/cm^2^ und Stunde. Unter der Annahme einer einstündigen Exposition von 2000 cm^2^ Hautoberfläche würde dies Aufnahmemengen von 14 400 bzw. 1468 mg entsprechen.

### 3.2 Metabolismus

Ethylformiat hydrolysiert enzymatisch zu Ameisensäure und Ethanol (Greim 1997 b). Es ist plausibel, dass auch durch die nasale Carboxylesterase die gleichen Metaboliten entstehen. Die Hydrolyse dürfte dabei ähnlich schnell erfolgen wie beim strukturverwandten Ethylacetat (Hartwig und MAK Commission 2017).

Im Vergleich zu Nagern kann sich bei Primaten Ameisensäure anreichern. Ethanol wird über Acetaldehyd und Essigsäure hauptsächlich zu Kohlendioxid oxidiert. Bei 100 ml Ethylformiat/m^3^ ist keine Akkumulation dieser Metaboliten und keine metabolische Azidose zu erwarten (Greim 1997 b).

## 4 Erfahrungen beim Menschen

Im Folgenden werden Untersuchungen nach einmaliger Exposition, zur Reizwirkung und zur sensibilisierenden Wirkung beim Menschen dargestellt. Zu allen übrigen Endpunkten liegen keine neuen Studien vor.

### Einmalige Exposition

In einer Probandenstudie wurden mit standardisierten psychophysischen Messungen die Geruchs- und Irritationsschwellen für Ethylformiat bestimmt (van Thriel et al. 2006). Die Irritationsschwelle wurde als Lateralisierungsschwelle bestimmt, die auf einer eindeutigen Stimulation intranasaler Nervenendigungen des Nervus trigeminus basiert. Deren Stimulation kann – im Gegensatz zu der von olfaktorischen Rezeptoren – einer Nasenseite zugeordnet, d. h. lateralisiert werden (u. a. Hummel 2000; Kobal et al. 1989). In der alters- und geschlechtsgeschichteten Stichprobe von 72 Probanden (jeweils 18 Männer und 18 Frauen in den beiden Altersgruppen zwischen 18 und 35 Jahren sowie ≥ 45 Jahre) mit intakter Geruchsfunktion lag der Median der Geruchsschwelle bei 30 ml/m^3^, der der Lateralisierungsschwelle bei 13 800 ml/m^3^. Alters- und Geschlechtsunterschiede wurden nicht beobachtet. Im Vergleich dazu liegen die Lateralisierungsschwellen für Ethylacetat und Ethylacrylat bei 1229 bzw. 4,15 ml/m^3^ und deren MAK-Werte betragen 200 ml/m^3^ bzw. 2 ml/m^3^. Da chemosensorische Schwellenwerte auf sehr kurzen Expositionszeiten beruhen (im Sekundenbereich), lassen sich jedoch keine Aussagen über mögliche Gesundheitseffekte bei länger andauernden Expositionen machen (van Thriel et al. 2006). Die Reizwirkung von Ethylformiat beim Menschen ist somit geringer als die der beiden anderen Ester.

Außerdem wurden die Zusammenhänge zwischen Expositionshöhe und Geruchs- oder Reizempfindungen ermittelt. Dazu wurde die Intensität der trigeminalen und olfaktorischen Wahrnehmung an 48 Probanden (ebenfalls alters- und geschlechtsgeschichtet) untersucht. Von den Probanden wurden die olfaktorischen Wahrnehmungen („Geruchsintensität“, „Lästigkeit“ und „Ekel erregend“) sowie die trigeminal vermittelten Wahrnehmungen (unter anderem „brennend“, „stechend“, „scharf“) mit der jeweiligen Intensität von „kaum wahrnehmbar“ bis „stärkste vorstellbare Wahrnehmung/Intensität“ bewertet. Es wurden neun verschiedene Konzentrationen von 59 bis 7200 ml/m^3^ in aufsteigender Reihenfolge getestet, wobei jede der Verdünnungsstufen fünfmal für jeweils zwei Sekunden den Probanden präsentiert wurde. Die höchste der untersuchten Konzentrationen liegt unterhalb der Lateralisierungsschwelle (Median: 13 800 ml/m^3^), die niedrigste liegt oberhalb der Geruchsschwelle (Median: 30 ml/m^3^). Im Konzentrationsbereich um die Geruchsschwelle (19–71 ml/m^3^, entspricht unterem (25 %) und oberem (75 %) Quartil) wurden schwache bis mäßige olfaktorische und kaum wahrnehmbare trigeminale Empfindungen angegeben. Erst bei sehr hohen Konzentrationen kam es zu stärkeren Empfindungen. Im Bereich der Lateralisierungsschwelle (7240–21 800 ml/m^3^, entspricht unterem und oberem Quartil) liegen die Bewertungen am oberen Ende der Skala und werden als stärkste vorstellbare Lästigkeit und stärkstes vorstellbares Stechen beschrieben. Alters- und Geschlechtsunterschiede wurden nicht beobachtet. Die Autoren kommen zu dem Schluss, dass die chemosensorische Potenz von Ethylformiat bei Einhaltung des MAK-Wertes von 100 ml/m^3^ relativ gering ist (Blaszkewicz et al. 2007; van Thriel et al. 2006).

In einer Studie von 1931 führten 330 ml Ethylformiat/m^3^ zu leichten Reizungen der Augen und zu rasch zunehmenden Reizungen der Nase, die noch nach vier Stunden in verstärktem Maße anhielten, 10 500 ml Ethylformiat/m^3^ bewirkten mittelschwere, jedoch progressive Irritationen der Augen und der Nase (Flury und Zernik 1931 in Greim 1997 b). In der Übersichtsarbeit von Ruth et al. (1986) ist eine Irritationsschwelle von 990 mg/m^3^ (321 ml/m^3^) aufgeführt, die sich wahrscheinlich auf die Studie von 1931 bezieht. In der Literatur findet sich ferner die standardisierte Geruchsschwelle mit 18 ml/m^3^ (van Thriel et al. 2006).

### Wirkung auf Haut und Schleimhäute

Die 48-stündige okklusive Applikation von 4 % Ethylformiat in Vaseline führte bei Freiwilligen zu keinen Reizeffekten auf der Haut (k. w. A.; Opdyke 1978). Die Originalstudie liegt nicht vor.

### Allergene Wirkung

Es liegen keine verwertbaren Daten vor. Lediglich eine sekundär zitierte Untersuchung aus den 1960er Jahren weist auf einen Maximierungstest an 23 Freiwilligen hin, welcher negativ verlief (k. w. A.; Opdyke 1978).

## 5 Tierexperimentelle Befunde und In-vitro-Untersuchungen

### 5.1 Akute Toxizität

Hierzu liegen keine neuen Daten vor.

Die 20-minütige Exposition von Mäusen und Katzen führte ab 5000 ml Ethylformiat/m^3^ zu Augenreizungen. Bei Katzen bewirkte die 75-minütige Exposition gegen 10 000 ml/m^3^ tiefe Narkose und führte nach 90 Minuten zum Tod. Für Ratten und Hunde war eine vierstündige Exposition gegen 8000 bzw. 10 000 ml/m^3^ letal (Greim 1997 b).

Die orale LD_50_ wurde an Ratten mit 1850 mg/kg KG, an Meerschweinchen mit 1110 mg/kg KG und an Kaninchen mit 2075 mg/kg KG ermittelt. Für Kaninchen lag die dermale LD_50_ bei über 20 ml/kg KG (> 18 000 mg/kg KG) (Greim 1997 b).

### 5.2 Subakute, subchronische und chronische Toxizität

#### 5.2.1 Inhalative Aufnahme

In einer 90-Tage-Inhalationsstudie nach OECD-Prüfrichtlinie 413 wurden jeweils zehn Sprague-Dawley-Ratten pro Geschlecht und Gruppe an sechs Stunden pro Tag, fünf Tage pro Woche gegen 0, 66, 330 oder 1320 ml Ethylformiat/m^3^ Ganzkörper-exponiert. Ab 330 ml/m^3^ wurden bei den weiblichen Tieren Degenerationen des olfaktorischen Epithels beobachtet (siehe Tabelle 1), bei 1320 ml/m^3^ auch bei den männlichen Tieren. Bei beiden Geschlechtern traten in dieser Expositionsgruppe Plattenepithelmetaplasien des olfaktorischen Epithels auf; ferner verminderte Futteraufnahme, verzögerte Körpergewichtsentwicklung (ohne Zunahme der Befunde mit der Zeit, Vergleich von vier und 13 Wochen) sowie reduzierte lokomotorische Aktivität, die nach Ende der jeweiligen Expositionen reversibel war (Lee und Kim 2017). Für weibliche Tiere beträgt die lokale NOAEC 66 ml/m^3^, die für männliche Tiere 330 ml/m^3^. Bei beiden Geschlechtern ist die systemische NOAEC 330 ml/m^3^, die systemische LOAEC 1320 ml/m^3^.

**Tab. 1:** 90-Tage-Inhalationsstudie mit Ethylformiat an Ratten (Lee und Kim 2017)

**Spezies, Stamm, Anzahl pro Gruppe**	**Exposition**	**Befunde**
**Ratte**, Sprague-Dawley, 10 ♂, 10 ♀	**90 d**, 0, 66, 330, 1320 ml/m^3^, 6 h/d, 5 d/Wo, **OECD-Prüfrichtlinie 413**	**66 ml/m^3^**: ♀: **NOAEC_lokal_**; **330 ml/m^3^**: ♂: **NOAEC_lokal_**; ♂/♀: **NOAEC_systemisch_**; ♀: Ketonkörper im Urin ↑, Degeneration des olfaktorischen Epithels (2/10, n. sign.); **1320 ml/m^3^**: ♂/♀: Futterverbrauch (♂ ab 1., ♀ ab 3. Wo) u. KG-Entwicklung ↓ ab 1. Wo, abs./rel. Nebennierengewicht ↑, Ketonkörper im Urin ↑, Degeneration des olfaktorischen Epithels (♂ 9/10, ♀ 10/10), Plattenepithelmetaplasie im olfaktorischen Epithel (♂ 2/10, ♀ 1/10), lokomotorische Aktivität ↓ (nach Expositionsende reversibel), ♂: abs. Thymusgewicht ↓, Urobilinogen im Urin ↑, Hämoglobin u. Hämatokrit im Blut ↑, Calcium im Serum ↓, ♀: abs./rel. Thymusgewicht ↓, Retikulozyten im Blut ↓, Triglyceride im Serum ↓

abs.: absolutes; d: Tage; h: Stunden; KG: Körpergewicht; n. sign.: nicht statistisch signifikant; rel.: relatives; Wo: Woche

#### 5.2.2 Orale Aufnahme

Hierzu liegen keine validen Studien vor.

#### 5.2.3 Dermale Aufnahme

Hierzu liegen keine validen Studien vor.

In der im Abschnitt 5.7 beschriebenen dermalen Kanzerogenitätsstudie an Mäusen (S-Strain), in der die Tiere einmal pro Woche, zehn Wochen lang mit 2760 mg Ethylformiat/kg KG und Tag (unverdünnt) und Crotonöl (einmal pro Woche, 18 Wochen lang, Beginn: drei Tage nach erster Ethylformiat-Gabe) behandelt wurden, wurden keine systemischen oder lokalen Effekte berichtet. Die Applikation erfolgte offen. Drei Tage nach der ersten und zweiten Behandlung mit Ethylformiat wurden keine epidermalen Hyperplasien beobachtet (Roe und Salaman 1955).

### 5.3 Wirkung auf Haut und Schleimhäute

#### 5.3.1 Haut

Unverdünntes Ethylformiat wurde auf die Ohren (20 Minuten lang) und den Rücken (1, 5, 15 oder 20 Minuten lang) von Kaninchen appliziert (k. w. A.). Die Ablesung erfolgte nach 24 Stunden und nach acht Tagen. Nur am Ohr war nach 24 Stunden eine leichte Rötung zu beobachten, die reversibel war (ECHA 2022).

Die Reizwirkung nach 24-stündiger Applikation von unverdünntem Ethylformiat auf die geschorene Haut von je fünf männlichen Neuseeländer-Kaninchen wurde mit 1/10 („the least visible capillary injection“) bewertet (Smyth et al. 1954). Unverdünntes Ethylformiat wirkte auch bei 24-stündiger okklusiver Applikation weder auf intakter noch auf abradierter Kaninchenhaut reizend (k. w. A.; ECB 2000; ECHA 2022).

#### 5.3.2 Auge

Etwa 50 mg unverdünntes Ethylformiat führte eine Stunde nach Instillation am Kaninchenauge zu leichter bis starker Rötung, die auch nach 24 Stunden noch als leicht bewertet wurde (k. w. A.; ECHA 2022).

Laut einer Studie aus den 1960er Jahren wirkt Ethylformiat am Kaninchenauge ätzend („corneal necrosis“) mit 4/10 Bewertungspunkten. Werden durch 0,5 ml der unverdünnten Testsubstanz nur kleine Bereiche der Cornea nekrotisiert, wird die Punktzahl 1 vergeben, bei „severe burn“ durch 0,005 ml die Punktzahl 5, und 10 bei 0,5 ml einer 1%igen Lösung (Smyth et al. 1954).

#### 5.3.3 Fazit

Ethylformiat wirkt nicht reizend an der Kaninchenhaut und reizend am Kaninchenauge. Der Stoff ist nach dem global harmonisierten System nicht als hautreizend, aber als augenreizend in die Kategorie 2 eingestuft (ECHA 2022).

### 5.4 Allergene Wirkung

Hierzu liegen keine Daten vor.

### 5.5 Reproduktionstoxizität

#### 5.5.1 Fertilität

Es liegen keine Generationen- oder Fertilitätsstudien vor.

In der bereits beschriebenen 90-Tage-Inhalationsstudie wurden jeweils zehn Sprague-Dawley-Ratten pro Geschlecht und Gruppe gegen 0, 66, 330 oder 1320 ml Ethylformiat/m^3^ an sechs Stunden pro Tag, fünf Tage pro Woche ganzkörperexponiert. Bei männlichen und weiblichen Tieren untersuchte Reproduktionsorgane (Hoden, Nebenhoden, Prostata, Samenbläschen bzw. Ovarien, Uterus) waren bei der höchsten Konzentration von 1320 ml Ethylformiat/m^3^ ohne auffällige makroskopische und mikroskopische Befunde (siehe Abschnitt 5.2; Lee und Kim 2017).

#### 5.5.2 Entwicklungstoxizität

Hierzu liegen keine Studien mit Ethylformiat an Nagern vor.

Die Injektion von bis zu 25 mg Ethylformiat pro Ei in den Dottersack oder die Luftzelle zu Beginn oder 96 Stunden nach Beginn der Bebrütung führte bei Hühnerembryos zu keinen strukturellen oder funktionellen entwicklungstoxischen Effekten (Verrett et al. 1980).

Im Folgenden werden die Daten zu den primären Metaboliten von Ethylformiat, den Hydrolyseprodukten Ethanol und Ameisensäure, dargestellt.

Für **Ethanol** ist in der Begründung von 1998 und im Nachtrag von 2018 ausführlich dargestellt, dass die fruchtschädigen﻿de Wirkung nach oraler Aufnahme bei Mensch und Tier nachgewiesen ist (Greim 1998; Hartwig und MAK Com﻿mis﻿sion 2018).

**Ameisensäure** führt in vitro bei Ratten- und Mäuseembryonenkulturen ab 11,8 mmol/l zu erhöhter Mortalität, die auf die Absenkung des pH-Wertes zurückgeführt wird (Greim 1997 a).

### 5.6 Genotoxizität

#### 5.6.1 In vitro

Bereits in der Begründung 1997 wird über einen negativen Salmonella-Mutagenitätstest mit den Stämmen TA1535, TA1537 und TA1538 in Konzentrationen bis zu 5 % mit und ohne Zusatz eines metabolischen Aktivierungssystems aus Mäuse-, Ratten- oder Affenleber-Homogenaten berichtet. Auch mit Saccharomyces cerevisiae verlief ein Mutagenitätstest negativ (Greim 1997 b).

Ethylformiat war auch in Präinkubationstests mit Konzentrationen bis zu 10 000 µg/Platte in den Salmonella-typhimurium-Stämmen TA98 und TA100 sowie E. coli WP2 uvrA pKM101 in An- und Abwesenheit eines metabolischen Aktivierungssystems nicht mutagen (NTP 2018).

In Saccharomyces cerevisiae wurden durch Ethylformiat weder mitotische Aneuploidien oder Rekombinationen noch Punktmutationen ausgelöst (ACGIH 2012; Health Council of the Netherlands 2002).

#### 5.6.2 In vivo

Hierzu liegen keine validen Daten vor.

In einem Abstract wird von einem Anstieg an Letalmutationen in befruchteten Eiern von Drosophila melanogaster nach Exposition gegen Ethylformiat-Dampf berichtet (k. w. A.; ACGIH 2012).

### 5.7 Kanzerogenität

Nach topischer offener Applikation von 0,3 ml unverdünntem Ethylformiat (276 mg pro Applikation, einmal pro Woche, zehn Wochen lang) und Behandlung mit Crotonöl (einmal pro Woche, 18 Wochen lang, Beginn: drei Tage nach erster Ethylformiat-Gabe) induzierte Ethylformiat keine Hauttumoren bei 20 Mäusen (S-Strain). In der Kontrollgruppe, die 18 Wochen lang nur Crotonöl erhielt, trat bei einer von 20 Mäusen ein Hauttumor auf. Somit war Ethylformiat kein Initiator in diesem Testsystem. Von 13 überlebenden Tieren, die Ethylformiat und Crotonöl erhalten hatten, wiesen eine Woche nach Beendigung der Crotonöl-Gabe sechs insgesamt 15 Lungenadenome auf. Im Vergleich dazu hatten fünf von 15 untersuchten Mäusen der Crotonöl-Gruppe, die insgesamt 72 Wochen behandelt wurden, Lungenadenome (Roe und Salaman 1955). Da Ethylformiat in vitro nicht mutagen ist, für strukturähnliche Verbindungen (z. B. Ethylacetat und Ethanol bei Inhalation) kein Verdacht auf eine kanzerogene Wirkung besteht und außerdem die Exposition dermal und nur einmal pro Woche erfolgte, ist die deutliche Lungenadenom-Inzidenz nicht plausibel. Eine unbehandelte Kontrollgruppe fehlte.

Valide Langzeitstudien zur Kanzerogenität von Ethylformiat liegen weiterhin nicht vor.

## 6 Bewertung

Kritische Effekte von Ethylformiat sind die lokale Reizwirkung an Auge und Nase des Menschen und am olfaktorischen Epithel von Ratten sowie die im Tierversuch bei hohen Konzentrationen beobachteten akuten zentralnervösen Effekte.

**MAK-Wert. **Da die Probandenstudien mit Ethylformiat nur mit Expositionszeiten im Sekundenbereich durchgeführt wurden, sind sie nicht für die Ableitung eines MAK-Wertes geeignet.

**MAK-Wert-Ableitung über lokale Wirkung**: Für Ethylformiat liegt eine 90-Tage-Inhalationsstudie an Ratten vor, in der die lokale NOAEC 66 ml/m^3^ beträgt (Lee und Kim 2017). Bei der nächsthöheren Konzentration von 330 ml/m^3^ kommt es zu Degenerationen im olfaktorischen Epithel bei 2/10 weiblichen und bei keinem männlichen Tier. Ausgehend von der lokalen NOAEC von 66 ml/m^3^ ergibt sich unter Berücksichtigung einer möglichen Zunahme der Effekte bei chronischer Exposition (1:2) eine Konzentration von 33 ml/m^3^. Da die Carboxylesterase-Aktivität im olfaktorischen Epithel, durch die Ethylformiat zu Ethanol und Ameisensäure hydrolysiert wird, bei Nagern und Mensch in etwa gleich ist (Hartwig und MAK Commission 2017), muss keine Spezies-Extrapolation erfolgen. Unter Anwendung des Preferred Value Approach würde ein MAK-Wert von 10 ml/m^3^ resultieren. Im Vergleich dazu konnte in einer 90-Tage-Studie mit **Ethylacetat** an Ratten keine lokale NOAEC bestimmt werden, da acht von 20 Tieren bereits bei der niedrigsten Konzentration von 350 ml/m^3^ minimale Degenerationen des olfaktorischen Nasenepithels aufwiesen. Die Reizwirkung von Ethylformiat ist also geringer als die von Ethy﻿lacetat (2/20 Tiere gegenüber 8/20). Die Lateralisierungsschwellen beim Menschen für Ethylformiat und Ethylacetat lagen bei 13 800 bzw. 1229 ml/m^3^ (van Thriel et al. 2006). Das heißt, auch beim Menschen ist die Reizwirkung von Ethylformiat geringer als die von Ethylacetat. Der MAK-Wert von Ethy﻿lacetat von 200 ml/m^3^ wurde aus mehreren Humanstudien abgeleitet. Deshalb wird für die lokale Wirkung von Ethylformi﻿at wie bei Ethylacetat der MAK-Wert nicht aus der Rattenstudie abgeleitet. In einer alten, sehr schlecht dokumentierten Probandenstudie wurde die Konzentration von 330 ml/m^3^ als reizend bewertet (Greim 1997 b). Trotz der schlecht belegten Reizwirkung beim Menschen wird diese Konzentration als LOAEC angenommen und daraus eine NAEC von 110 ml/m^3^ (1:3) extrapoliert. Der bisherige MAK-Wert von 100 ml/m^3^ kann beibehalten werden. Der MAK-Wert von Ethylformiat liegt höher als der seines Metaboliten Ameisensäure (5 ml/m^3^). Bei Inhalation von Ameisensäure trifft diese direkt auf das Zielgewebe, das olfaktorische Epithel. Nach Inhalation des Esters muss dieser zunächst durch die Carboxylesterase metabolisiert werden, wodurch die Reizwirkung des Esters geringer ist als die der Säure. Daher kann der MAK-Wert für die lokale Wirkung von Ethylformiat höher sein als der von Ameisensäure. Dies ist analog zu den Verhältnissen bei Ethylacetat (MAK-Wert 200 ml/m^3^) und Essigsäure (MAK-Wert 10 ml/m^3^).

**MAK-Wert-Ableitung über systemische Wirkung**: In der bereits oben erwähnten 90-Tage-Inhalationsstudie an Ratten beträgt die systemische NOAEC 330 ml Ethylformiat/m^3^ (Lee und Kim 2017). Bei der nächsthöheren Konzentration von 1320 ml/m^3^ wiesen die Tiere (reversible) reduzierte lokomotorische Aktivität sowie verminderte Futteraufnah﻿me und verzögerte Körpergewichtsentwicklung auf. Bei der verminderten Aktivität ist ein pränarkotischer Effekt zu vermuten, der konzentrations- aber nicht zeitabhängig ist. Reduzierte Futteraufnahme sowie die verzögerte Körpergewichtsentwicklung verstärken sich mit der Zeit nicht, sodass bei der Ableitung des MAK-Wertes nicht mit einer Verstärkung der Effekte bei chronischer Exposition gerechnet werden muss. Ausgehend von der NOAEC von 330 ml/m^3^ unter Berücksichtigung, dass diese aus einer tierexperimentellen Untersuchung stammt (1:2) und des erhöhten Atemvolumens am Arbeitsplatz (1:2) resultiert eine Konzentration von 83 ml/m^3^. Da die LOAEC viermal so hoch wie die NOAEC war, könnte die tatsächliche NAEC auch etwas höher liegen. Somit würde diese Studie dem MAK-Wert von 100 ml/m^3^ nicht widersprechen und dieser auch die systemische Wirkung abdecken.

**Plausibilitätsprüfung**: Der MAK-Wert des strukturanalogen **Methylformiats** in Höhe von 50 ml/m^3^ ist aus einer Probandenstudie aufgrund von neuropsychologischen Veränderungen abgeleitet worden mit Unterstützung von Arbeitsplatzstudien und den darin beobachteten zentralnervösen Effekten. Die NOAEC für diese Wirkungen liegt bei 100 ml/m^3^. Als kritischer Metabolit wird die Ameisensäure angesehen, die für das Auftreten der metabolischen Azidose verantwortlich gemacht wird (Hartwig und MAK Commission 2019). Da aus einem Mol Methylformiat zwei Mol Ameisensäure entstehen, aus einem Mol Ethylformiat ein Mol Ameisensäure gebildet wird, ist ein MAK-Wert für Ethylformiat in Höhe von 100 ml/m^3^ konsistent mit dem MAK-Wert von Methylformiat, der die Hälfte beträgt.

Der bestehende MAK-Wert für Ethylformiat in Höhe von 100 ml/m^3^ schützt damit auch vor der systemischen Wirkung.

**Spitzenbegrenzung. **Da die lokale NOAEC in der 90-Tage-Inhalationsstudie niedriger liegt als die systemische NOAEC, erfolgt die Zuordnung von Ethylformiat zur Spitzenbegrenzungs-Kategorie I. Aufgrund der in einer Probandenstudie als reizend bewerteten Konzentration von 330 ml/m^3^ wird der Überschreitungsfaktor 1 beibehalten.

**Fruchtschädigende Wirkung. **Untersuchungen zur entwicklungstoxischen Wirkung von Ethylformiat liegen weiterhin nicht vor. Wie in der Begründung von 1997 (Greim 1997 b) bereits dargestellt, ist bei 100 ml/m^3^ weder mit einer Akkumulation der Metaboliten Ethanol oder Ameisensäure noch mit einer metabolischen Azidose zu rechnen. Die pulmonale Retention von Ethanol betrug beim Menschen für Expositionskonzentrationen von 5800 bis 10 000 ml/m^3^ und für Ventilationsraten von 7 bis 25 l/min durchschnittlich 62 % (Greim 1998). Da der Metabolit Ethanol bei einem MAK-Wert von 200 ml/m^3^ der Schwangerschaftsgruppe C zugeordnet ist, ist eine fruchtschädigende Wirkung von Ethylformiat bei Einhaltung des MAK-Wertes von 100 ml/m^3^ für Ethylformiat nicht zu erwarten, selbst wenn eine 100%ige inhalative Resorption und Hydrolyse für Ethylformiat angenommen wird. Auch für die bei Einhaltung des MAK-Werts gebildete Konzentration an Ameisensäure ist nicht zu erwarten, dass sie zu reproduktionstoxischen Effekten führt (Greim 1997 a). Ethylformiat bleibt daher weiterhin der Schwangerschaftsgruppe C zugeordnet.

**Krebserzeugende Wirkung. **Für die Bewertung des kanzerogenen Potenzials von Ethylformiat liegen keine geeigneten Studien vor. Aus der Struktur ergibt sich kein entsprechender Verdacht. Ethylformiat wird deshalb weiterhin nicht in eine Kategorie für Kanzerogene eingestuft.

**Keimzellmutagene Wirkung. **Valide Studien an Keimzellen liegen nicht vor. Ethylformiat ist in Bakterien und Hefen nicht mutagen. Weitere Daten liegen nicht vor. Es erfolgt keine Einstufung in eine Kategorie für Keimzellmutagene.

**Hautresorption. **Der Stoff war bisher in Analogie zu Methylformiat mit „H“ markiert. Die Modelle zur Hautpenetration ergeben eine Aufnahmemenge von mindestens ca. 1500 mg unter Standardbedingungen (2000 cm^2^, eine Stunde). Die systemische NAEC für den Menschen ist 100 ml/m^3^ (310 mg/m^3^) (siehe „MAK-Wert-Ableitung über systemische Wirkung"). Bei Annahme einer 100%igen inhalativen Resorption und einem Atemvolumen von 10 m^3^ beträgt die systemisch tolerable Menge 3100 mg. Die über die Haut aufgenommene Menge ist mehr als 25 % davon und daher bleibt Ethylformiat mit „H“ markiert.

**Sensibilisierende Wirkung. **Es liegen keine verwertbaren klinischen Daten vor. Tierexperimentelle Untersuchungen oder Studien mit tierversuchsfreien Alternativ-Verfahren fehlen. Strukturell besteht kein Verdacht für eine sensibilisierende Eigenschaft. Ethylformiat wird daher weiterhin nicht mit „Sh“ markiert. Zur atemwegssensibilisierenden Wirkung von Ethylformiat liegen keine Daten vor, sodass weiterhin keine Markierung mit „Sa“ erfolgt.
